# Hepatitis B Virus Reactivation Induced by Infliximab Administration in a Patient with Crohn's Disease

**DOI:** 10.1155/2013/461879

**Published:** 2013-06-09

**Authors:** Yuka Miyake, Aki Hasebe, Tetsuya Tanihira, Akiko Shiraishi, Yusuke Imai, Haruka Tatsukawa, Hiroka Yamago, Hiromasa Nakahara, Yuko Shimizu, Keiko Ninomiya, Atsushi Hiraoka, Hideki Miyata, Tomoyuki Ninomiya, Kojiro Michitaka

**Affiliations:** ^1^Gastroenterology Center, Ehime Prefectural Central Hospital, Kasugamachi 83, Matsuyama, Ehime 790-0024, Japan; ^2^Internal Medicine, Saiseikai Imabari Hospital, Imabari 799-1502, Japan

## Abstract

A 47-year-old man diagnosed with Crohn's disease was treated with infliximab. He tested negative for hepatitis B surface antigen (HBsAg) and hepatitis B surface antibody (anti-HBs) but positive for anti-HB core antibody (anti-HBc). He tested positive for hepatitis B virus (HBV-) DNA 3 months after treatment and was administered entecavir. HBV-DNA test showed negative results 1 month later. ALT was persistently within the normal range, and HBV-DNA was persistently negative thereafter despite the continuation of infliximab every 8 weeks. In our hospital, 14 patients with inflammatory bowel disease, who tested negative for HBsAg, were treated with infliximab; 2 of them tested positive for anti-HBs and/or anti-HBc, and HBV reactivation was observed in 1 patient (the present patient). The present case and these findings highlight that careful follow-up is needed in patients with inflammatory bowel disease treated with infliximab who test positive for anti-HBc and/or anti-HBs.

## 1. Introduction

Crohn's disease is a chronic and intractable inflammatory disorder of the gastrointestinal tract [1]. Many types of therapy have been introduced to treat Crohn's disease, such as nutritional therapy, 5-aminosalicylic acid drugs, corticosteroids, azathioprine, and 6-mercaptopurine [2–6]. Monoclonal antibodies against tumor necrosis factor alpha (anti-TNF*α*), such as infliximab and adalimumab, have recently been used to treat inflammatory bowel disease [7–9]. Short- and long-term anti-TNF-*α* therapies in Crohn's disease are generally well tolerated. However, clinicians must be vigilant for the occurrence of infrequent but serious events [10].

 Immunosuppressive therapy may induce the reactivation of hepatitis B virus (HBV), not only in patients in an inactive hepatitis B surface antigen (HBsAg) carrier state, but, in resolved patients as well. HBV reactivation in HBV-resolved patients may cause hepatitis (i.e., de novo hepatitis). Moreover, hepatitis B reactivation due to immunosuppressive therapy sometimes progresses to severe hepatitis, and several fatal cases have been reported [11, 12]. 

Here, we report the case of a patient with Crohn's disease who tested negative for HBsAg and positive for anti-HB core antibody (anti-HBc) and exhibited HBV reactivation during treatment with anti-TNF-*α* antibody (infliximab).

## 2. Case Report

**Figure 1:**
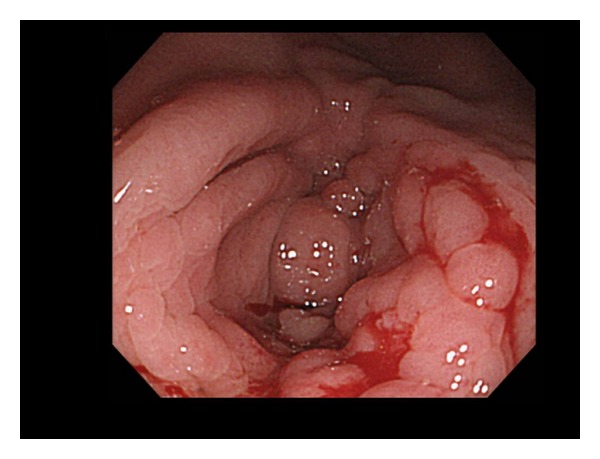
Photograph of lower endoscopic examination. A longitudinal ulcer scar, a cobblestone appearance, and stenosis were observed at the hepatic flexure.

**Figure 2:**
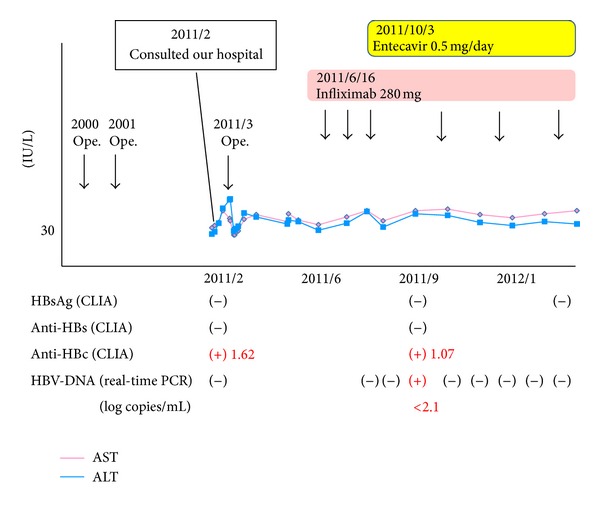
Clinical course. HBV-DNA and ALT levels were screened every month after the start of infliximab.

A 47-year-old man, with a history of abdominal operations because of perforation of the ileum and ileus in 2000 and 2001, respectively, and diagnosed with Crohn's disease histologically in 2000, was admitted to our hospital because of abdominal pain. He was diagnosed with intestinal stricture of the ascending colon due to Crohn's disease (Figure 1). He was treated by resection of the ascending colon, followed by treatment with anti-TNF-*α* (i.e., infliximab). The patient was negative for HBsAg, hepatitis B surface antibody (anti-HBs), and HBV-DNA, but positive for anti-HBc upon admission (Table 1). He was examined for HBV-DNA monthly after the start of infliximab. HBV-DNA was assayed by quantitative real-time polymerase chain reaction. Infliximab at 5 mg/kg body weight was administered. Infliximab was scheduled to be administered 2 and 6 weeks after the first administration, and then every 8 weeks. His symptoms improved after the beginning of treatment. He tested positive for HBV-DNA 3 months after the beginning of treatment (after 3 injections) (Figure 2). Although he had no symptoms and his alanine aminotransferase (ALT) level was within the normal range, he was administered entecavir according to the Japanese Guidelines [13]. HBV-DNA test showed negative results 1 month later. ALT was persistently within the normal range and HBV-DNA was persistently negative thereafter despite the continuation of infliximab every 8 weeks.

In our hospital, 15 patients with inflammatory bowel diseases (including Behçet's disease) have been treated with infliximab. A list of patients and their HBV markers is shown in Table 1. One patient was positive for HBsAg; he was diagnosed as having an inactive HBsAg carrier state. Entecavir was administered from the beginning of infliximab administration (Table 2). Among the other 14 patients who tested negative for HBsAg, 2 tested positive for anti-HBc and/or anti-HBs. One was positive for both anti-HBs and anti-HBc, and the other (the present patient) was positive for only anti-HBc. Both patients were assayed for HBV-DNA monthly, and HBV-DNA reactivation was observed in the present patient.

## 3. Discussion

 HBV reactivation is one of the major problems in patients receiving immunosuppressive therapy or chemotherapy against malignant tumors. Approximately 350 million and 3 billion people worldwide are infected with HBV chronically and transiently, respectively. They have a risk of reactivation of HBV if they receive these therapies. The American Association for the Study of Liver Diseases Practice Guidelines indicate that the rates of HBV reactivation in transiently infected people (i.e., those negative for HBsAg and positive for anti-HBc and/or anti-HBs) receiving mild, moderate, and intense immunosuppressive therapy are 1.0–2.7%, 12%, and 14–20%, respectively, [14]. 

Recently, anti-TNF-*α* has been widely used in patients with rheumatoid arthritis, psoriatic arthritis, ulcerative colitis, Crohn's disease, and ankylosing spondylitis. The risk of HBV reactivation under anti-TNF*α* administration has been studied, but the actual risk remains controversial. Several reports emphasize the risk of reactivation. Urata et al. studied HBV reactivation in patients with rheumatoid arthritis who received various therapies, and found that 17 of 135 patients experienced reactivation; moreover, the use of biologic agents was significantly more frequent in patients who developed HBV-DNA reactivation than in patients who did not [15]. Another report indicates that the risk of reactivation is higher with infliximab administration than with other anti-TNF-*α* agents [16]. On the other hand, several reports indicate the safety of anti-TNF-*α* agents [17]. Tamori et al. report that none of the 21 patients with rheumatoid arthritis who received infliximab experienced reactivation [18]. The majority of these studies were performed on patients with rheumatoid arthritis, and the information regarding its risk in patients with inflammatory bowel disease is scarce. The immune state may be different between patients with rheumatoid arthritis and those with inflammatory bowel disease; therefore, it is needed to clarify this issue in patients with inflammatory bowel disease. There are a few reports that described the risk of severe hepatitis in patients with Crohn's disease treated with anti-TNF-*α* agents; however, information was still insufficient [19–23]. In the present study, 1 out of 2 patients with Crohn's disease with resolved hepatitis exhibited reactivation. The exact rates of reactivation due to specific anti-TNF-*α* agents (e.g., infliximab, adalimumab, and etanercept) should be clarified in the future.

Kato et al. studied which HBV markers are related to HBV reactivation in patients who received immunosuppressive therapy and found that a low titer of baseline anti-HBs may increase the risk [22]. In the present study, 2 patients displayed markers for resolved hepatitis B; one patient tested positive for anti-HBc and negative for anti-HBs experienced reactivation and the other patient was positive for both anti-HBc and anti-HBs did not experience reactivation. The present findings are concordant with those of Kato et al.

The present patient was administered entecavir immediately when HBV-DNA became positive with very low level. It has been recommended by the Japanese Guidelines [13] that entecavir should be administrated when HBV-DNA became positive in patients treated with immunosuppressive agents, but it is still controversial whether entecavir should be administrated immediately when HBV-DNA became positive with a very low level (or positive only by qualitative assay). This issue should be further investigated.

 There is a shortage of information regarding the prevalence of HBV markers in patients with inflammatory bowel disease. Reports from France and Spain indicate that the prevalence of HBV markers in patients with inflammatory bowel disease is similar to that in the general population [24, 25]. In the present study, 3 out of 15 patients displayed markers of present or past HBV infection; 1 was positive for HBsAg, and 2 (13.3%) were positive for anti-HBc and/or anti-HBs and negative for HBsAg. There are too few patients in the present study to adequately discuss this issue. However, it must be noted that a considerable percentage of patients with inflammatory bowel disease display HBV markers. Therefore, HBV markers including anti-HBs and anti-HBc should be examined in patients who are scheduled for anti-TNF-*α* treatment, and careful follow-up is needed after the beginning of anti-TNF-*α* administrationif any HBV markers are positive. 

## Figures and Tables

**Table 1:** Laboratory data on admission.

Urine			BUN	12.4	mg/dL
Protein	(−)		Creatinine	1.34	mg/dL
Sugar	(−)		Na	136	mEq/L
Blood	(−)		K	4.3	mEq/L
Urobil.	(±)		Cl	105	mEq/L
			Ca	8.1	mg/dL
WBC	4300	/*μ*L	Amylase	169	IU/L
RBC	359 × 10^4^	/*μ*L			
Hb	11.5	g/dL	CRP	3.75	mg/dL
Ht	33.1	%			
Platelet	20.1 × 10^4^	/*μ*L	PT	89.1	%
			PT (INR)	1.05	
T. protein	6.4	g/dL			
Alb.	3.4	g/dL	HBsAg	(−)	CLIA
T. bil.	0.3	mg/dL	Anti-HBs	(−)	CLIA
AST	42	IU/L	Anti-HBc	(+) 1.62	CLIA
ALT	45	IU/L	HBV-DNA	(−)	Real-time PCR
ALP	348	IU/L	Anti-HCV	(−)	CLIA
CHE	238	IU/L			
*γ*-GTP	56	IU/L			

PCR: polymerase chain reaction; CLIA: chemiluminescence immunoassay.

**Table 2:** HBV markers in patients treated with infliximab.

Age	Sex		HBsAg	Anti-HBs	Anti-HBc s/co	HBV reactivation	Entecavir
47	F	Crohn	(+)				(+)
54	F	Crohn	(−)	(+)	(+) 6.41	(−)	(−)
47	M	Crohn	(−)	(−)	(+) 1.62	(+)	(+)
55	F	Crohn	(−)	(−)	(−) 0.9		
64	F	UC	(−)	(−)	(−) 0.09		
19	M	Crohn	(−)	(−)	(−) 0.05		
34	M	Crohn	(−)	(−)	(−) 0.11		
52	F	UC	(−)	(−)	(−) 0.07		
43	M	Crohn	(−)	(−)	(−) 0.09		
38	M	Behçet	(−)	(−)	(−) 0.11		
52	M	UC	(−)	(−)	(−) 0.08		
18	F	Crohn	(−)	(−)	(−) 0.05		
42	M	Crohn	(−)	(−)	(−) 0.15		
41	M	UC	(−)	(−)	(−) 0.07		
23	M	Crohn	(−)	(−)	(−) 0.14		

Crohn: Crohn's disease, UC: ulcerative colitis, Behçet: Behçet's disease, M: male, F: female.
